# RNAi-Based Approaches for Pancreatic Cancer Therapy

**DOI:** 10.3390/pharmaceutics13101638

**Published:** 2021-10-08

**Authors:** Min Ju Kim, Hyeyoun Chang, Gihoon Nam, Youngji Ko, Sun Hwa Kim, Thomas M. Roberts, Ju Hee Ryu

**Affiliations:** 1 Department of Cancer Biology, Dana-Farber Cancer Institute, Boston, MA 02115, USA; simple5336@gmail.com (M.J.K.); chang.hyeyoun@gmail.com (H.C.); ghnam@shiftbio.net (G.N.); YoungJ_Ko@dfci.harvard.edu (Y.K.); 2 Center for Theragnosis, Biomedical Research Institute, Korea Institute of Science and Technology (KIST), Seoul 02792, Korea; sunkim@kist.re.kr

**Keywords:** RNA interference, siRNA, miRNA, pancreatic cancer, nanocarrier

## Abstract

Pancreatic cancer is one of the most lethal forms of cancer, predicted to be the second leading cause of cancer-associated death by 2025. Despite intensive research for effective treatment strategies and novel anticancer drugs over the past decade, the overall patient survival rate remains low. RNA interference (RNAi) is capable of interfering with expression of specific genes and has emerged as a promising approach for pancreatic cancer because genetic aberrations and dysregulated signaling are the drivers for tumor formation and the stromal barrier to conventional therapy. Despite its therapeutic potential, RNA-based drugs have remaining hurdles such as poor tumor delivery and susceptibility to serum degradation, which could be overcome with the incorporation of nanocarriers for clinical applications. Here we summarize the use of small interfering RNA (siRNA) and microRNA (miRNA) in pancreatic cancer therapy in preclinical reports with approaches for targeting either the tumor or tumor microenvironment (TME) using various types of nanocarriers. In these studies, inhibition of oncogene expression and induction of a tumor suppressive response in cancer cells and surrounding immune cells in TME exhibited a strong anticancer effect in pancreatic cancer models. The review discusses the remaining challenges and prospective strategies suggesting the potential of RNAi-based therapeutics for pancreatic cancer.

## 1. Introduction

Pancreatic cancer is one of the most aggressive malignancies, ranking as the fourth leading cause of cancer-related deaths with a five-year survival rate of 8% [1]. If outcomes are not improved, pancreatic cancer is predicted to be the second leading cause of cancer-related mortality by 2025 [2]. With a minimal increase in patient survival rate in past four decades, pancreatic cancer is often discovered and diagnosed at advanced stage where metastasis have already taken asymptomatically, and limited treatment options are left [3]. Because of aggressive perineural and vascular local growth, chemotherapy, radiotherapy, and molecularly target therapies are better treatment options than surgical resection. Typically, chemotherapy elongates the life span only by 8 to 16 weeks, and there is an urgent need for new therapeutic options for pancreatic cancer patients [4]. In particular, pancreatic cancer is characterized both by complicated genetic mutations and epigenetic alterations and by development of a dense tumor microenvironment that together result in a low survival rate of just ~15–25% of patients who undergo surgical resection [5]. One of the unique histological features of pancreatic cancer is a dense fibrotic stroma surrounding the tumor that comprises around 80% of the whole tumor mass [6]. In addition to the early metastasis and robustly immunosuppressive TME, this protective stromal barrier and distorted tumor blood vessels contribute greatly to poor survival by increasing the difficulty of drug delivery and distribution [7,8,9,10]. In addition, the abnormal vasculature induces hypoxic environment in TME which contributes to resistance towards chemotherapy and immunotherapy [11,12]. Desmoplastic features with an abundance extracellular matrix are known to be developed by the cancer-associated pancreatic stellate cells (PSCs) in communication with the surrounding cells in the TME [13]. The PSCs that are normally quiescent in the healthy pancreas are recruited upon cancer cell communication to fuel the aggressive proliferation of tumors [14,15,16]. Given the clear importance of TME in tumorigenesis, approaches targeting specific features within TME have been investigated in addition to the focus on directly targeting tumor cells.

In recent decades, studies have delineated the genetic mutations unique to pancreatic cancer. For example, 94% of pancreatic tumors feature mutations along with mutations in the *TP53* (64%), *SMAD4* (21%), and *CDKN2A* (17%) tumor suppressor genes [17]. The largest challenge with the genetic aberrations of pancreatic cancer is that the *KRAS* oncogene has been ‘undruggable’ using the now conventional treatment approach of targeted chemical inhibitors [18]. Undruggable targets represent proteins that is pharmacologically incapable of being targeted due to elusive factors from molecular shapes and mechanism of action. For this reason, the potential to selectively inhibit undruggable genes with RNAi represents a promising strategy to halt pancreatic cancer progression. RNAi is a post-transcriptional mechanism that involves the inhibition of specific gene expression through promoting cleavage of the relevant messenger RNA (mRNA). After decades of investigation, in 2018 the first RNAi-based drug, Patisiran (ONPATTRO^®^) finally proved the potential of siRNA therapeutics as demonstrated by FDA approval and market entry [19]. In the following years, Givosiran (GIVLAARI^®^) and Lumasiran (OXLUMO^®^) were FDA approved in 2019 and 2020, embarking the era of RNAi-based oligo therapeutics. Thus, RNAi-based technology has presented an opportunity to regulate a set of tumor-promoting genes in cancer cells and other targets in TME. However, the clinical application of RNAi therapeutics is still hindered by poor delivery to the target organs and inefficient internalization into target cells. In this review, we discuss the prospects and challenges in RNAi-based approaches using various strategies of nanomedicine to target key genes in cancer cells or TME of pancreatic cancer. 

## 2. RNAi Targets for Pancreatic Cancer Therapy

**Figure 1:**
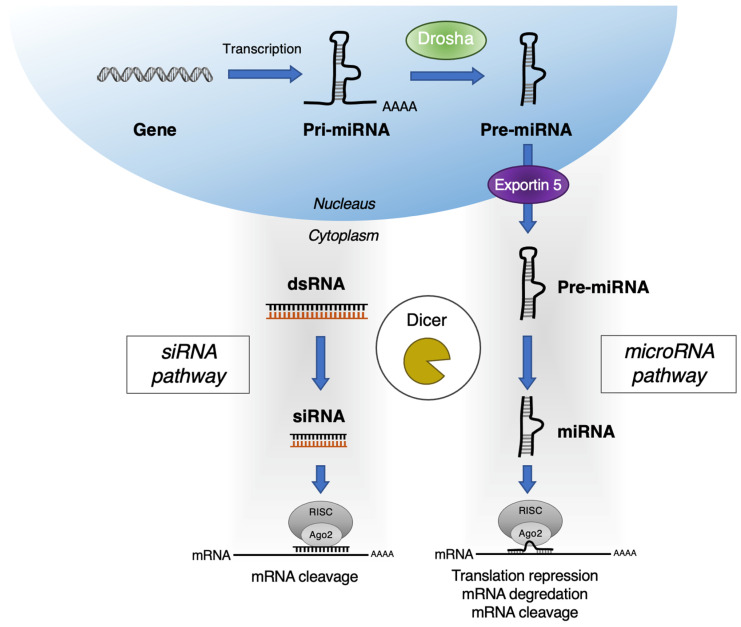
Gene silencing mechanism of siRNA and miRNA of RNAi.

RNAi is a regulatory mechanism using small regulatory RNAs to prevent translation of mRNA and regulate protein expression levels. As an intrinsic mechanism for post-transcription editing, RNAi comprises two major different types of RNA molecules, siRNA, and miRNA, generated via similar mechanisms involving the dicer protein (Figure 1). Both siRNA and miRNA are short non-coding RNA strands that can silence the target mRNA in sequence-specific manner, therefore offering the advantage of greater selectivity and expanding the inhibitory action for undruggable targets. Despite numerous challenges involving delivery, safety, and potency, RNAi-based drugs have shown therapeutic potential in rare genetic diseases and complex diseases such as cancer [20,21,22,23,24].

siRNA is a 20 to 25 base pairs double-stranded non-coding RNA that plays a key role in RNAi machinery. Once internalized in a cellular system, the siRNA forms an activated RNA-induced silencing complex to guide pairing with a target mRNA of complementary sequence resulting in cleavage and degradation [25,26]. The ability of siRNA to silence specific gene expression involved in diseases has led to a significant effort to use it as an alternative treatment option. Despite the breakthroughs in the generation of siRNA therapeutics beyond preclinical studies, siRNA delivery into cells at the disease site has remained the major hurdle in various clinical applications. The challenges include the negatively charged nature of siRNA, immediate degradation by the serum proteins, and fast clearance from the reticuloendothelial system [27]. Furthermore, the dense fibrotic stroma and vascular barriers of pancreatic cancer add an extra layer of difficulty in successfully delivering siRNA to the tumor and surrounding cells in TME. We will use this forum to introduce siRNA-based approaches to pancreatic cancer therapy using various reagents and strategies. 

miRNAs are 19–25 nucleotides long, endogenous non-coding RNAs found in living organisms as one of the key players in the RNAi machinery as shown in Figure 1 [28]. Similar to siRNA, miRNA interfere with the expression of specific proteins by binding to the mRNA strand. miRNA is a long bimolecular RNA duplex that partially binds to the untranslated region of multiple genes to interfere with the post-transcriptional expression. Contrary to the direct and specific inhibition of siRNA, miRNA often hinders the translation of several mRNAs via closely related binding sites in the 3′ regions of target mRNAs [29]. Compelling evidence has demonstrated that miRNA is involved in the cellular machinery and signaling contributing to the dysregulation of cancer cell growth [30]. miRNA may function as oncogenes or tumor suppressors interfering with a set of specific protein expression. The dysregulation of miRNA is known to be prevalent across cancer types, contributing to sustaining proliferative signaling, evading growth suppressors, resisting cell death, activating invasion, and inducing metastasis and angiogenesis. For this reason, miRNA-based therapeutics that inhibit the levels of oncogenic signaling or elevate tumor suppressor functionality have enormous potential [31].

### 2.1. Pancreatic Tumor Targets

Conventional treatment approaches are focused on the eradication of pancreatic cancer using chemotherapies. The potency of a of given procedure is related to its ability to effectively remove cancerous cells; however, many therapies often harm the normal organs causing major side effects in patients [32]. To overcome these drawbacks, targeted therapies have been advanced as the next generation of cancer therapies. The carcinogenesis of pancreatic ductal adenocarcinoma (PDAC), comprising 90% of the pancreatic cancers, involves progressive accumulation of key driver mutations, usually leading to the activation of the KRAS oncogene and loss of the tumor suppressor gene TP53, contributing to the histological evolution of pancreatic intraepithelial neoplasia to PDAC [33,34,35]. These genetic aberrations induce invasive adenocarcinoma and remodel the surrounding stromal structure into one more favorable for tumor formation. Because of the unique stromal feature and frequent oncogenic mutations, wide range of pancreatic cancer models exist for investigation for varying purposes as summarized in Table 1. 

Often, the genes discovered to be the novel cancer-related oncogenes that promote tumor progression are found to be ‘undruggable,’ since they are difficult to inhibit using small drug molecules [36]. Because of lack of suitable ligand binding sites or the presence of large protein-protein interaction interfaces, various oncogenes are relatively intractable using small-molecule inhibitors [37]. A notable undruggable target, mutationally activated KRAS, is present in >90% of PDAC and represents the most frequent (>90%) and the earliest genetic alternation, found even in low-grade pancreatic intraepithelial neoplasia 1A lesions [38,39]. Moreover, KRAS activation is also a poor prognostic factor resulting resistance to traditional treatments [5,39,40]. Genetically engineered mouse models also support the initiating role of KRAS mutation in PDAC and its involvement in progression to the malignancy [41,42]. When coupled with mutational inactivation of TP53 (TP53R175H), CDKN2A, or SMAD4, pancreatic intraepithelial neoplasia formation rapidly evolves into high-frequency occurrence of metastatic PDAC [41,43,44]. In recent decades, KRAS has been an intractable challenge as the therapeutic target with no targeted inhibitors. Recently a breakthrough was made with the generation of KRAS^G12C^ inhibitors which have proved effective in non-small cell lung cancer patients in phase I clinical trials; however, only 2% of the PDAC carry KRAS^G12C^ specific mutations rather than the common KRAS^G12D^ mutation. Thus, while KRAS^G12C^ inhibitors are a great proof of concept, their existence only increases the urgency to develop alternative KRAS-targeting moieties such as RNAi [45]. In addition to the prominent undruggable KRAS mutation, other aberrations in gene expression in PDAC also emphasize the need for RNAi mediated approach for therapy. Table 2 summarizes the reported siRNA therapeutics used in pancreatic cancer therapy by targeting various ‘undruggable’ genes. Most importantly, the incorporation of delivery agents such as nanoparticles and extracellular vesicles have facilitated the delivery of siRNA to cancer cells.

For miRNA, there are numerous evident data from in vitro models as well as from PDAC patient samples proving dysregulated levels of miRNA in pancreatic cancer [46]. From analyzing 95 miRNAs expression levels in pancreatic cancer tissues and cell lines, eight miRNAs—miR-196a, miR-190, miR-186, miR-221, miR-222, miR-200b, miR-15b, and miR-95—were significantly up-regulated in most pancreatic cancer samples. On the other hand, miR-148a, miR-217, miR-34a and miR-375 tumor suppressor miRNAs are frequently down-regulated in PDAC [47,48]. Over the past decade, extensive research efforts have been dedicated to investigating the underlying mechanism of chemoresistance. Along with dysregulation of various genes involved in key signaling pathways, such as phosphoinositide-3-kinase (PI3K), NF-κB, and hedgehog, miRNAs have also been demonstrated to modulate the expression associated with chemoresistance in PDAC [49]. For this reason, miRNA has gained attention as a useful biomarker for PDAC prognosis and as a target for therapeutic applications. Table 3 summarizes the miRNA-based approaches for pancreatic cancer therapy now in preclinical studies. 

**Table 2:** siRNA-based therapeutics in preclinical pancreatic cancer models.

Category	Delivery Vehicle	siRNA Target	Tumor Model	Drug Route	Combination Therapy	Reference
Lipid-based Nanoparticles	Lipid nanoparticle (LNPK15)	KRAS	MIA PaCa-2 s.c.	I.V.		[50]
	Lipoplex	KRAS	PANC1 s.c.	I.V.		[51]
	Lipoplex (Atu027)	PKN3	DanG orthotopic	I.V.		[52]
	Liposome	KRAS	PANC-1 s.c.	I.V.	Gemcitabine	[53]
Polymer-based Nanoparticles	Gold nanocluster siRNA (GNC-siRNA)	Nerve growth factor	PANC-1 s.c., orthotopic and PDX	I.T.		[54]
	Superparamagnetic iron oxide nanoparticles (siPLK1-StAv-SPIONs)	PLK1	6606PDA orthotopic [55]	I.V.		[56]
	Star polymeric nanoparticles different lengths of cationic PDMAEMA side-arms and varied amounts of POEGMA	βIII-tubulin	MiaPaCa-2 and HPAF-II orthotopic	I.T.		[57,58]
	Poly(ethylene glycol) and charge-conversional polymer (PEG-CCP)	VEGF	L1-Luc/TAg transgenic [59]	I.V.		[60,61]
	Local Drug EluteR, LODER (PLGA)	KRAS	PANC1-Luc or Capan1-Luc s.c., synograft, and orthotopic	I.T.		[62]
	PLGA/poloxamer	EPAS1	BxPC3 s.c.	I.T.		[63]
	Cholesterol-modified polymeric CXCR4 antagonist (PCX) nanoparticles	NCOA3	CD18/HPAF orthotopic	I.V.		[64]
	Cholesterol-modified polymeric CXCR4 antagonist (PCX) nanoparticles	KRAS	KPC8060 orthotopic	I.V. and I.P.		[65]
	BCPV	KRAS	MiaPaCa-2 s.c.	Peritumoral		[66]
	Folic acid (FA)-modified PEG-chitosan oligosaccharide lactate (COL) nanoparticles	ARHGEF4, CCDC88A, LAMTOR2, mTOR, NUP85, and WASF2	S2-013 orthotopic	I.V.		[67]
	PEGylated iRGD-guided tumor-penetrating nanocomplexes (TPN)	KRAS	KP D8-175 orthotopic from Pdx1-Cre; Krasþ/LSL-G12D; Trp53fl/ (KPC) mouse	I.V.		[68]
	poly(ethylene glycol)-block-poly-L-lysine (PEG-PLL)	KRAS	PANC-1 (mutant KrasGGT_GAT), BXPC-3 (KrasWT) s.c.	I.V.	Arsenic therapy	[69]
	Peptide-conjugated PSPG (PSPGP)	TR3	PANC-1 s.c.	I.V.	Paclitaxel	[70]
	Magnetic nanocarrier	PD-L1	PANC-02 syngeneic	I.V.	Gemcitabine	[71]
Extracellular vesicle	Exosome	KRAS	PANC-1 or BxPC-3/1) PANC-1, BxPC-3, or KPC689 orthotopic tumor mice model 2) KTC (Ptf1acre/+;LSL-Kras^G12D^/+;Tgfbr2lox/lox) genetically engineered mouse	I.P.		[72]
	Extracellular vesicle	Galectin-9	PANC-02 orthotopic	I.V.	Oxaliplatin	[73]

Abbreviations: PDMAEMA, Poly[2 (Dimethylamino)ethyl methacrylate]; POEGMA, Polyoligo(ethylene glycol) methyl ether methacrylate; PLGA, Poly lactic-co-glycolic acid; PSPG, G2 dendrimers through disulfide linkages; PEG, polyethylene glycol; RGD, arginylglycylaspartic acid; CXCR4, C-X-C chemokine receptor type 4; BCPV, Biodegradable charged polyester-based vector; PKN3, protein kinase N3; NGF, nerve growth factor; PLK1, polo-like kinase 1; VEGF, vascular endothelial growth factor; EPAS1, endothelial PAS domain protein 1; NCOA3, nuclear receptor coactivator 3; ARHGEF4, rho guanine nucleotide exchange factor 4; CCDC88A, coiled-coil domain containing 88a; LAMTOR2, late endosomal/lysosomal adaptor, MAPK, and mTOR activator 2; mTOR, mechanistic target of rapamycin; NUP85, nucleoporin 85; WASF2, Wiskott–Aldrich syndrome protein family member 2; PD-L1, programed death-ligand 1; s.c., subcutaneous; PDX, patient-derived xenograft; I.V., intravenous; I.P., intraperitoneal; I.T., intratumoral.

### 2.2. Targets in the Pancreatic Tumor Stroma and Immunosuppressive Microenvironment

The prominent stromal structure of PDAC consists of several cellular and acellular components, which together play a critical part in disease progression [83,84,85]. Although substantial research studies have focused on developing small-molecule or biological inhibitors directly targeting the cancer cells interest has more recently turned to agents targeting the tumor microenvironment. The key players of PDAC microenvironment are PSCs and cancer-associated fibroblasts responsible for the desmoplasia as well as various immune cells including immunosuppressive myeloid-derived suppressor cells, tumor-associated macrophages, and regulatory T cells [86]. The unique stromal barrier is often the cause of drug resistance while tumor induced alterations of the stroma have been suggested to facilitate tumor progression and proliferation. Thus, the concept of stomal reprogramming holds considerable promise [87].

There have been numerous siRNA targets in TME and stromal or immune cells to seek therapeutic effect for pancreatic cancer. One of the unique drivers of the dense stroma in PDAC are the activated PSCs. The PSCs are normally in a quiescent state functioning as a storage of vitamin A-rich lipid droplets [88]. During the initial development of PDAC, the PSCs are switched to an activated state losing the ability to produce lipid droplets and instead, producing an excessive amount of extracellular matrix components and proinflammatory chemokines [89]. The extracellular matrix production disrupts the normal parenchyma, leading to hypovascularity, and hypoxic conditions, which in turn further activates PSCs maintaining a hypoxia-fibrosis cycle [90]. Toll-like receptors are proteins that recognize foreign pathogen-associated molecular patterns and play a key role in the innate immune system. Recently, it has been shown that PSCs express a variety of toll-like receptors contributing to the activation of proinflammatory signaling pathways [91,92]. In particular, the toll-like receptor 4 is heavily involved in the stromal development in pancreatic cancer and has been advanced as a therapeutic target in PSCs [92]. Based on this, vitamin A-coupled liposomes carrying toll-like receptor 4-silencing small hairpin RNAs were used to target PSCs, reducing fibrosis by inducing mitochondrial apoptosis [93]. Ishiwatari et al. similarly used vitamin A-coupled liposomes (VA-lip) containing siRNA to induce collagen secretion by activated PSCs [94]. The siRNA targeting the collagen specific chaperone protein gp46 in VA-lip successfully inhibited the secretion of collagen and tissue collagenases, which dissolves the existing collagen matrix. Furthermore, Han et al. used a TME-responsive nanosystem to re-educate the PSCs to return to a quiescent state [95]. The nanosystem consists of polyethylene glycol-modified polyethyleneimine-coated gold nanoparticles to co-deliver all-*trans*-retinoic acid and siRNA targeting heat shock protein 47 (hsp47). The combination strategy successfully restored homeostatic stromal function and improved the efficacy of chemotherapy in stroma-rich tumor Another molecular target in the TME is the tumor-associated macrophages, of which depletion or reprogramming can induce immunogenic attack of the tumor cells [96]. Although various targets have been explored to reprogram or deplete tumor-associated macrophages, inhibition of the colony stimulating factor 1 (CSF1) or C-C Motif Chemokine Ligand 2 (CCL2) signaling have been particularly reported [97,98]. As one of the most abundant tumor-infiltrating leukocytes in all types of cancer, tumor-associated macrophages tend to stay in an alternative M2 state rather than the classically activated M1 state [99]. The M2 phase macrophage exhibit numerous tumor-promoting properties such as angiogenic signaling and restraining adaptive immune responses arising from the interactions of dendritic cells and CD^8+^ T cells. To deplete M2 macrophage, Qian et al. developed a dual-targeting nanoparticle (M2NPs), whose structure and function are controlled by two targeting moieties, an α-peptide coupled with an M2 macrophage binding protein for extremely specific targeting of M2 macrophage [100]. Upon loading of an anti-colony stimulating factor-1 receptor targeted siRNA, M2NPs showed higher affinity to M2-like tumor-associated macrophages than to tissue-resident macrophages resulting in the elimination of M2 macrophage, thus decreasing tumor size in cancer bearing mice. In a later study, colony stimulating factor-1 receptor silencing siRNA was co-delivered with PI3K inhibitor, BEZ-235 using an M2 tumor-associated macrophage targeting nanomicelle to elicit therapeutic immune responses against pancreatic cancer cells both in vitro and in tumor models [101]. The combination treatment switched the macrophage state from M2 to M1, thus, reducing tumor infiltration of myeloid-derived suppressor cells. The reprogramming of M2 tumor-associated macrophage activated antitumor immune responses and enhanced anti-pancreatic tumor effects in a Pan-02 pancreatic xenograft model.

## 3. RNAi Delivery Strategies for Pancreatic Cancer Therapy

### 3.1. Nanocarrier-Mediated RNAi Therapy

**Figure 2:**
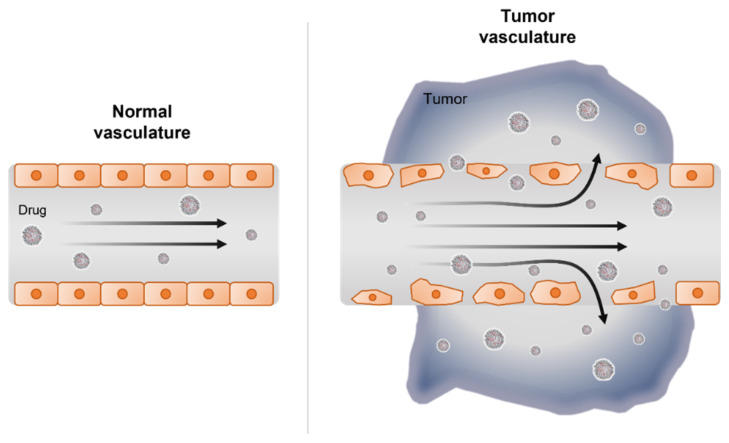
Drug delivery in vasculature of normal and tumor tissue.

Over the last several decades of nanotechnology advancement, several applications and products containing nanomaterials have become available in the field of medicine [102]. Nanotechnology for medical purposes has been termed nanomedicine and is defined as the use of nanomaterials for diagnosis, monitoring, prevention, and treatment of diseases [103]. Nanomaterials can be applied in nanomedicine largely in three areas: diagnosis, drug delivery, and regenerative medicine. As discussed earlier, the direct injection of naked, unmodified siRNA was not found to be effective as siRNA will faces challenges of RNA degradation, short half-life, and circulation time, and poor biodistribution on systemic injection. Direct chemical modifications on the 2′ carbon of the RNA ribose ring have been shown to prolong the half-life of siRNA without altering its functionality [104]. However, the modified siRNA has still shown discouraging results in clinical trials due to poor delivery to target tissues. For this reason, nanocarrier-mediated strategies have emerged as tool for therapy by acting as a protective delivery agent for siRNA. To provide the best opportunity to penetrate and accumulate within the cancer cells of the tumor, the size of delivery agent should range from 10 to 200 nm [105]. This size enables the nanoparticle to passively localize in the tumor sites via ‘enhanced permeability and retention effect’ (EPR effect) which arises from the leaky tumor vascular structure [106] (Figure 2). Nanoparticles that are over 10 nm in size are too large to penetrate healthy vessels and tight-gap junctions [107]. Several passively targeted nanomedicines—including Doxil™, Abraxane™, Marqibo™, DaunoXome™, and Onivyde™—are currently in clinical use, suggesting that the EPR effect can drive therapeutic outcomes [108]. Although EPR effect is the underlying mechanism for nanomedicine, active targeting of nanoparticles with ligands and modalities are also being used as strategies to increase the target ability of RNAi therapeutics. Active targeting can significantly increase the quantity of drug delivered to the target cell compared to free drug or passively target nanosystems [109]. Ligands that target transferrin receptors or nicotinic acetylcholine receptors or even antibodies greatly enhance targeting of nanomedicine. In addition to nanoparticles, naturally occurring extracellular vesicles have also gained attention as a possible RNAi delivery agent also capable of biological communication with the tumor by harnessing cytokines and signaling proteins. Here, we will discuss some recent studies have demonstrated the potential of siRNA therapeutics using nanoparticles and extracellular vesicles for effective siRNA delivery and release into the tumor cell.

#### 3.1.1. Lipid-Based Nanoparticle Delivery

Lipid-based nanoparticles are siRNA delivery agents consisting of pH-sensitive cationic lipid components. The lipids are amphiphilic and capable of self-assembly into micelles or liposomes in conjunction with siRNA via electronic charge interactions under acidic conditions [110]. Lipid-based nanoparticles such as liposomes are a suitable carrier for both small-molecule drug and nucleic acid delivery because of their excellent biocompatibility, biodegradability, low toxicity and immunity, structural flexibility, and ease of mass-production [111]. Decades after the discovery of RNAi, Patisiran (ONTATTRO™), which is a lipid-based nanoparticle formulated with siRNA targeting transthyretin, was FDA approved in 2018 as the first siRNA drug for the treatment of transthyretin-type amyloid polyneuropathy [112]. Because *KRAS* is so frequently mutated and active in cancer, especially in pancreatic cancer, *KRAS* is often the molecular target of siRNA therapeutics.

In 2019, Sasayama et al. generated a lipid nanoparticle formulation (LNPK15) to deliver KRAS-targeting siRNA to MIA PaCa-2 cells in vitro and in tumors in vivo achieving food protein knock down suggesting antitumor efficacy [50]. The group developed a PEGylated lipid-based nanoparticle named LNPK15 using two novel cationic lipids, SST-01 and SST-31, encapsulating KRAS siRNA that showed long circulation and efficient siRNA delivery to tumor sites. The *KRAS*-targeting LNPK15 showed strong antitumor efficacy in tumor xenograft mice of MIA PaCa-2 pancreatic cancer although the in vitro knock down activity of lipid-based nanoparticle was rather weak compared to its effect in tumors. Although PEGylation with 3.3% 1,2-distearoyl-sn-glycero-3-phosphatidylethanolamine-N-(polyethylene glycol-2000) (PEG-DSPE) provides benefit in terms of long durations, it could be disadvantageous for intracellular delivery and endosomal escape of siRNA, which explains the lower activity in cell lines. The in vitro activity was rescued with lipase treatment for lipid nanoparticle hydrolysis for enhanced knock down of *KRAS*.

Rao et al. have demonstrated *KRAS* down-regulation by a lipoplex encoding a short hairpin RNA via systemic injection in a pancreatic cancer model [51]. The lipoplex contained a plasmid encoding a short hairpin RNA that executes selective silencing of the mutated KRAS gene with tumor cell entry facilitated by facilitated by fusogenic liposomes. The bifunctional short hairpin RNA design is designed for more rapid silencing, higher efficacy, and greater durability of siRNA action compared to conventional short hairpin RNA [113]. The lipoplex containing the *KRAS*-targeting bi-short hairpin RNA was systemically injected in female athymic Nu/Nu mice bearing PANC1 xenografts and showed successful delivery. In addition to the *KRAS* mutation as therapeutic target for RNAi, other genes such as protein kinase N3 have been investigated as targets for siRNA in pancreatic cancer. Kaufmann’s group generated a liposomal formulation Atu027) for protein kinase N3 siRNA delivery from neutral fusogenic and polyethylene glycol-modified lipid components (for improved pharmacokinetic properties, cellular uptake, and efficient siRNA release in cells [52]. The siRNA-lipoplexes were capable of accumulating in tumor endothelial cells in vivo and halted tumor growth in two different xenograft mouse models [114]. As protein kinase N3 has previous been identified as a downstream effector of the PI3K signaling pathway, reduction of protein kinase N3 expression with Atu027 resulted in changes in tumor lymphatic vasculature, suggesting an anti-lymph angiogenesis therapeutic strategy for pancreatic cancer [115].

In addition to the demonstration of therapeutic potential of siRNA, miRNA has also been investigated in pancreatic cancer therapy. Pramanik et al. selected miR-34a and miR-143/145 for PDAC therapy based on their involvement in cancer stem cell survival and expression of KRAS and effectors. The miRNAs were formulated in lipid-based nanovector and intravenously injected in MiaPaCa-2 subcutaneous and orthotopic models where they effected tumor growth inhibition and increased apoptosis [82]. The success of miRNA-based therapeutics in preclinical studies has led some candidates to enter human clinical trials for cancer and other diseases [116,117,118]. The first ever miRNA-based clinical trial was with the miR-122 targeting called drug Miravirsen, which features the use of an LNA-based miRNA inhibitor, and has gone on to Phase II with for Hepatitis C virus infected liver cells [119]. Another miRNA mimicking drug MRX34, a lipid-based nanoparticle, was tested in clinical trials to treat advanced solid tumors [120]. However, the clinical trials involving miRNA-based drugs have largely been terminated because of severe adverse events arising from immune responses after treatments [121]. The two major discontinued miRNA-drug clinical trial results indicated that more than 30 genes in immunogenic and cytokine signaling pathways were identified to be affected by the miRNA machinery [122]. This results in the immune-related adverse events in phase I studies of miRNA-based drug clinical trials.

#### 3.1.2. Metal Nanoparticle USE for Delivery

For centuries, the noble metal gold has been studied first as a bulk material and now impacting nanomedicine [123]. In particular, gold nanoparticles (AuNPs) have shown important roles in the vaccine field as both an adjuvant and a carrier, by reducing toxicity, and increasing stability for storage [124]. Outside of vaccine applications, Lei et al. have shown that AuNPs are capable of siRNA delivery in vivo as nanocarriers for pancreatic tumor models [54]. As the nervous microenvironment has been recognized as a novel niche for PDAC progression and metastasis, nerve growth factor (NGF) and brain derived growth factor (BDGF) are rising targets for pancreatic cancer [125]. The gold nanocluster-assisted delivery of NGF targeting siRNA (GNC-siRNA) allowed efficient NGF gene silencing and pancreatic tumor treatment in subcutaneous, orthotopic, and patient-derived xenograft models. To add the biomimetic property, the AuNP nanoflowers (Au@BSA) were coated with bovine serum albumin (BSA) and conjugated with survivin-targeting siRNA for efficient delivery and inhibition of gene expression in BxPC-3 pancreatic cancer cells [126]. Other than AuNPs, iron oxide nanoparticles have also gained attention as a stable agent for thermal and immunotherapies for cancer [127]. Mahajan et al. developed superparamagnetic iron oxide nanoparticles coupled with siRNA directed against polo-like kinase-1 (PLK1), serving a dual purpose by both delivering siRNA to the tumor and facilitating non-invasive assessment of targeting efficiency via molecular imaging [56]. With uMUC1-specific ligand, EPPT1 and the myristoylated polyarginine peptide ligand and dextran, superparamagnetic iron oxide nanoparticle uptake was specific to pancreatic cancer and induced a drastic decrease in tumor volume in a syngeneic orthotopic model driven by PLK1 silencing. In addition to published reports of siRNA delivery using AuNPs, other inorganic nanoparticles such as graphene oxide and carbon nanotubes use were demonstrated in pancreatic cancer cells and models. Feng et al. developed a multi-functionalized monolayer graphene oxide as delivery agent for HDAC1 and KRAS siRNA in MIA PaCa-2 pancreatic cancer cells [128]. The dual gene silencing induced apoptosis, proliferation inhibition and cell cycle arrest and when treated in combination with near infrared light thermotherapy showed tumor volume growth inhibition for pancreatic in vivo models. Furthermore, Anderson et al. formulated a functionalized single walled carbon nanotubes as a siRNA carrier for in vitro gene therapy [129]. With KRAS siRNA in the carbon nanotube, PANC-1 cells showed great knockdown of KRAS gene expression.

#### 3.1.3. Polymer-Based Nanoparticle Delivery

McCarroll et al. has developed a star-shaped polymeric nanoparticle for delivery of siRNA into pancreatic cancer cells in vitro and in vivo. The star polymers are cost-effective nanoparticles that self-assemble with siRNA to produce a small (<50 nm) delivery system [58]. These star polymers, containing different lengths of cationic poly(dimethylaminoethyl methacrylate) side-arms and varied amounts of poly[oligo(ethylene glycol) methyl ether methacrylate], delivered siRNA targeting *β*III-tubulin to orthotopic pancreatic tumors [57]. Using this approach *β*III-tubulin expression was silenced by 80% and tumor growth progression was delayed when star polymers were administered in orthotopic models. Often, polyethylene glycol coating offers nanoparticles increased compatibility with aqueous environments. prolonging circulation time and reducing immunogenicity.

However, there has been biosafety issues from PEG recognizing antibodies contributing to immune responses and mitigation of therapeutic efficacy of PEGylated drugs. Despite these concerns, these nanoparticles have shown improved efficacy with polyethylene glycol fabrication when systemically administered [130]. Furthermore, Kataoka’s group have developed a polyethylene glycol coated calcium phosphate hybrid micelle system for siRNA delivery in pancreatic cancer [60,61]. First, the PEG-*b*-charge-conversional polymer/calcium phosphate hybrid micelles were systemically administered to EL1-Luc/TAg transgenic mice carrying luciferase targeting siRNA to ensure RNAi activity. As vascular endothelial growth factor (VEGF) is a key pro-angiogenic molecule in tumor progression, VEGF specific siRNA was then therapeutically administered with the help of the hybrid micelle. After systemic administration of purified nanoparticles, the reduction of expression level VEGF mRNA was evaluated in tumor tissues and found to be correlated with the pancreatic tumor growth rates [60]. Because of major issues concerning toxicity, safety, and clearance of inorganic nanoparticles, biodegradable and biocompatible organic materials have been sought out as a nanocarrier option for various drugs [131]. Poly (lactic-co-glycolic acid) (PLGA) is one of the most investigated biodegradable polymeric nanoparticles, and already has won FDA approval for medical applications [132]. Zorde Khvalevsky et al. synthesized a local prolonged siRNA delivery system, siG12D-LODER™, with PLGA for protection and gradual release of drug over time [62]. An siRNA against the mutated KRAS^G12D^ was encapsulated in LODER and implanted in a pancreatic xenograft and an orthotopic model, leading to decreased KRAS levels, as well as inhibition of cell proliferation and epithelial-mesenchymal transition over 155-day period. The group later injected the siRNA encapsulated LODER into tumors of locally advanced pancreatic cancer patients in combination with chemotherapy in a clinical trial study. Pan et al. also designed a PLGA/poloxamer nanoparticle loaded with EPAS1 siRNA and tested it on BxPC-3 pancreatic cancer cells in a mouse model of pancreatic cancer [133]. The endothelial PAS domain protein, also known as hypoxia-inducible factor-2, is a transcription factor known to regulate tumor cell adaption to hypoxic microenvironments and to accelerate metastatic spread [63]. The study demonstrated that the PLGA/poloxamer nanoparticle successfully inhibited VEGF and CD34 expression, therefore halting the growth of pancreatic tumors.

Oupicky’s group developed a different type of polyplex for nucleic acid delivery using cholesterol-modified polymeric CXCR4 antagonist nanoparticles to improve pancreatic cancer therapy [64,65]. The nuclear receptor coactivator-3 (NCOA3) is a critical modulator of musin expression that is critical in pancreatic cancer progression. The siRNA targeting NCOA3 was formulated along with a cholesterol-modified polymeric CXCR4 antagonist in a dual-function polyplex for delivery to tumors to inhibit CXCR4 chemokine receptor to treat metastatic pancreatic cancer [64]. Additionally, triple targeted nanoparticles featuring the CXCR4 antagonist for cancer stromal blockade action, as well as anti-miR-210 and siRNA targeting mutated *KRAS* were developed. The triple action-nanoparticle modulated the desmoplastic TME via inactivating PSCs and promoting infiltration of cytotoxic T cells [65].

Another organic material used for siRNA delivery is a biodegradable charged polyester-based vector which has demonstrated no significant toxicities on blood vessels, tissues, or organs even at high dosage [134]. When *KRAS*-targeting siRNA is formulated in this delivery vector and peritumorally injected in pancreatic xenograft models, significant decreases in tumor growth and local invasion were observed [66]. Many polysaccharide-derived nanoparticles such as chitosan nanoparticles are being investigated for nucleic acid drug delivery across diseases [135]. In particular, chitosan oligosaccharide lactate can be assembled with folic acid and polyethylene glycol into nanoparticles that are capable of encapsulating siRNA and intravenously localizing in tumors in pancreatic orthotopic models [67]. This formulated nanoparticle containing siRNA silencing genes *LAMTOR2, mTOR,* and *NUP85* have shown clinical potential by inhibiting retroperitoneal invasion and dissemination, therefore improving the prognosis of the mice. Overall, these studies indicate promising strategies of nanoparticle usage for siRNA therapeutics for pancreatic cancer therapy.

Other than the lipid and polymeric nanoparticles, peptides have also been investigated as siRNA delivery materials due to their biocompatibility and biodegradability for minimal toxicity [136]. In addition, peptides also have intrinsic property to act as ligands for targeting specific receptors. An arginine-glycine-aspartic acid-peptide is well-known to specifically bind to the integrin receptors overexpressed on the surface of cancerous cells [137]. Bhatia’s group formulated arginine-glycine-aspartic acid peptides with polyethylene glycol to encapsulate siRNA to generate a tumor-penetrating nanocomplex [68]. When a KRAS siRNA was intravenously delivered by this tumor-penetrating nanocomplex into a genetically engineered PDAC mouse model, the growth of pancreatic tumors arising from allografted KP D8-175 cells were significantly delayed. Similarly, pancreatic tumor inhibition was seen when miR-34a was delivered in nanocomplexes formulated with a tumor-targeting and penetrating bifunctional CC9 peptide [81]. The nanoparticle facilitated the cellular uptake of miR-34a and induced cell cycle arrest and blocked angiogenesis in xenograft model using PANC-1 cells. Another type of tumor suppressor miRNA, miR-150 was administered to pancreatic cancer cells in a PLGA-based nanoformulation incorporating polyethyleneimine. The cells showed a significant decrease in MUC4 expression and downstream signaling proteins such as human epidermal growth factor receptor 2 (hEGFR2), therefore reducing tumor growth, clonogenicity, motility, and invasion [74]. Due to these and other successes, the application of various nanoparticles is still being investigated in preclinical and clinical trials for pancreatic cancer therapy.

#### 3.1.4. Extracellular Vesicle-Mediated Delivery

Exosomes or extracellular vesicles are roughly 100 nm spherical vesicles, which are secreted by all cells. Initially, exosomes were considered to be garbage bags for cells [138]. However, recent findings demonstrating that exosomes contain a variety of bioactive molecules reflecting parental cells and play a crucial role in cell-cell signaling, have drawn much attention to exosome research [139]. In addition, exosomes exhibit many advantages as delivery systems including low immunogenicity, multi-functionality, and disease-targetability [140]. Notably, exosomes can be internalized by cells via a fusion mechanism, leading to efficient delivery of drugs into the target cell cytoplasm [141]. Some reports have indicated that exosomes expressing viral fusogen fused significantly with the target cell membrane in acidic conditions compared with native exosomes [142]. Notably, Liu et al. showed that viral fusogen decorated and programed death-ligand 1 siRNA-loaded, macrophage-derived extracellular vesicles substantially elevated tumor-specific CD8^+^ T cell immune responses in CT26 tumor-bearing mice through efficient programed death-ligand 1 gene silencing [143]. Although packing drug candidates into extracellular vesicles is still challenging, these findings indicate that the exosome has great potential as a drug delivery platform due to strengths mentioned above [144].

Several studies suggested that extracellular vesicles can effectively transfer RNAi into the cancer cells, eliciting substantial antitumor effects. For example, extracellular vesicles containing miRNA-134 enhanced the therapeutic effects of anti-heat shock protein 90 drugs in breast cancer [145]. Similarly, glucose-regulated protein 78 (GRP78) siRNA-loaded extracellular vesicles could sensitize resistant tumor cells to sorafenib [146]. Another study found that extracellular vesicle delivery of let-7a miRNA had potent antitumor effects on breast cancer cells breast cancer cells, and that extracellular vesicles expressing GE11 or AS1411, to target epidermal growth factor or nucleolin, respectively, on breast cancer cells, could enhance the effects of let-7a miRNA by enhancing delivery of their payload to the tumor cells [117,147]. Furthermore, taking advantage of fact that extracellular vesicles can penetrate the blood-brain barrier via transcytosis, systemic administration of extracellular vesicles harboring miRNA-21, miRNA-164b, miRNA-124a, or vesicular endothelial growth factor siRNA significantly retarded brain tumor growth, improving survival [148,149,150,151].

There have also been attempts to treat PDAC using exosomal siRNA. Kalluri et al. reported that mesenchymal stem cell-derived exosomes loaded with KRAS^G12D^ siRNA (termed iExosomes) provoked effective antitumor responses in pancreatic cancer models [72]. They also found that iExosomes showed a long half-life due to high expression of exosomal CD47, facilitating evasion of immunological clearance. The production protocol of iExosomes has been successfully established to generate clinical grade-exosomes that have entered phase 1 clinical trials for the treatment of PDAC [ClinicalTrials.gov identifier: NCT03608631] [152]. Zhou et al. recently developed engineered exosomes loaded with galectin-9 siRNA to alleviate immunosuppressive properties of tumor-associated macrophages [73]. Co-delivery of galectin-9 siRNA and the immunogenic chemotherapy oxaliplatin by exosomes elicited successful tumor control via immune activation. Collectively, these studies indicate that exosome-mediated gene silencing could be attractive for the treatment of PDAC.

### 3.2. Combination Therapy

Many studies have shown that the combination of siRNA and other therapeutic agents can exhibit synergistic effects to overcome multiple drug resistance by enhancing both silencing and chemotherapy activity [153,154]. Continuous administration of inhibitors often leads to the development of resistance via acquired mutations or epigenetic alterations. In combination therapy, the approach is designed to reverse the acquired genetic or epigenetic mechanism with the RNAi drugs instead of additional chemical inhibitors which result in increased toxicity. There have been numerous reports on therapeutic efficacy when existing therapeutic agents are combined with the concomitant use of RNAi drugs [155]. There is also evidence indicating that a combination strategy can also reduce the side effects of individual therapeutics by enhancing the therapeutic index [156].

As one of the most common cancer types with very low survival rate, pancreatic cancer is uniquely identified by its dense surrounding tumor microenvironment that limits the therapeutic effect of existing drugs. Both the dense stroma and acquired resistance to conventional therapies are triggered from dysregulated expression of cancer-associated genes. To target these key genes, RNAi-based approaches have gained attention both to elicit direct antitumor effects and to alter the nature of tumor microenvironment to remove barriers for chemotherapeutic action. Thus, several studies have sought to achieve antitumor therapeutic effects using KRAS siRNA in combination with chemotherapies and other small-molecule drugs. Zeng et al. reported that the combination of KRAS siRNA and arsenic trioxide effectively inhibited pancreatic tumor growth by exerting a synergistic effect of siRNA-induced cell cycle blockage and arsenic-induced apoptosis [69]. Wang et al. showed the combination of KRAS siRNA and gemcitabine exhibited improved inhibition of cell proliferation, cell cycle arrest, increased apoptosis, and suppression of tumor progression without any toxicity. In addition, they demonstrated that the anticancer effect of KRAS siRNA contributes to the low IC50 value of siRNA/gemcitabine-loaded liposomes [53]. Golan et al. tested that a biodegradable implant termed siG12D-LODER™ loaded with siRNA drug against KRAS^G12D^ and gemcitabine for pancreatic cancer. Of the 15 patients, 12 patients did not show tumor progression, 10 out of 12 patients showed stable disease, and 2 patients had partial response, yielding an increased average patient survival rate [157]. In addition, several attempts have been made to knock down several other genes highly associated with pancreatic cancer. Li et al. used a peptide-binding nanovehicle for co-delivery of paclitaxel and siRNA targeting TR3, an orphan nuclear receptor, finding that the co-delivery system exhibited a higher loading efficiency of siRNA, markedly inhibited tumor growth, and induced cancer cell apoptosis [70]. McCarroll et al. also identified βIII-tubulin as a novel mediator for chemoresistance and metastases in pancreatic cancer and used siRNA to increase chemosensitivity [158]. When βIII-tubulin expression is inhibited in orthotopic pancreatic cancer model in the presence of gemcitabine, tumor growth, and metastases were reduced in vivo. Additionally, siRNAs have been used to alter the immunosuppressive microenvironment and to reduce the stromal physical barrier to improve antibody or chemotherapeutic drug actions [159]. Yoo et al. combined an siRNA targeting the programed death-ligand 1 in a magnetic nanocarrier with gemcitabine to activate immune responses against tumor cells while noninvasively monitoring the therapeutic response via magnetic resonance imaging [71].

Currently, miRNA-based drugs are also being investigated in combination with chemotherapeutic drugs to enhance therapeutic efficacy in pancreatic cancer [160]. Although the chemotherapeutic gemcitabine remains a cornerstone of the PDAC treatment, patients acquire resistance within weeks of treatment [161]. To overcome the resistance, several novel therapeutic approaches as well as new entrapment designs with nanoparticles are currently being investigated. Patel et al. encapsulated miR-155 in extracellular vesicles from gemcitabine-resistant pancreatic cancer cells to reduce deoxycytidine kinase (gemcitabine-metabolizing gene) and increase superoxide dismutase 2 and catalase (ROS-detoxifying genes) expression in the presence of chemotherapy [75]. The deoxycytidine kinase reduction by miR-155 delivered by the extracellular vesicles led to abrogation of acquired resistance of gemcitabine in pancreatic cancer cells. Another miRNA that was combined with gemcitabine for pancreatic cancer treatment is miR-345 which was delivered in polymeric dual delivery nanoscale device [80]. The temperature and pH-responsive pentablock copolymer system enhanced/restored the miR-345 levels, making xenograft pancreatic cancer tumors more susceptible to gemcitabine. Immunohistochemical analysis revealed that the miR-345 induced significant down-regulation of desmoplastic reaction, as well as improving SHH, Gli-1, MUC4, and Ki67 levels. Because oncogenic miRNAs are responsible for triggering the cancerous features, antisense oligonucleotides are used to inhibit the activity of commonly dysregulated miRNAs [162]. Among numerous oncogenic miRNAs, miR-21 is one of the earliest identified and most abundant cancer-promoting miRNAs and hence has been targeted in cancer [163]. Li et al. developed a combined therapy using an miR-21 antisense oligonucleotide and gemcitabine co-delivered by a nanoparticle made from polyethylene glycol-polyethyleneimine-magnetic iron oxide [78]. With a coating of anti-CD44v6 single-chain variable fragment for targeted delivery, the miR-21 antisense oligonucleotide and gemcitabine dual-treatment nanoparticle up-regulated programed cell death protein 4 and phosphatase and tensin homolog (PTEN) expression, thus suppressing epithelial-mesenchymal transition, cell proliferation, migration, and invasion of pancreatic cancer cells. This combination therapy also showed a synergistic antitumor effect in a MIA PaCa-2 pancreatic xenograft model.

Other than gemcitabine, doxorubicin, and the hedgehog inhibitor, GDC-0944 have been combined with miRNA for pancreatic cancer therapy. Chen et al. developed a new chimeric peptide composed of plectin-1 for PDAC-specific targeting coupled with arginine-rich RNA-binding motifs for miR-212 delivery [79]. The reduction of USP9X expression by miR-212 enhanced the therapeutic effect of doxorubicin to induce apoptosis and autophagy of PDAC cells in vitro as well as in patient-derived pancreatic xenograft model. Kumar et al. selected miR-let7b as a therapeutic target because of inherently low expression miR-let7b in PDAC tumors with elevated hedgehog levels [164]. Using mPEG-block-poly(2-methyl-2-carboxyl-propylenecarbonate-graft-dodecanol-graft-tetraethylene-pentamine copolymer, miR-let7b and the hedgehog inhibitor, GDC-0944 were co-formulated into micelles, which inhibited tumor growth of an ectopic xenograft model by triggering apoptosis. Instead of combination with chemotherapy, miR-21 and miR-221 antisense oligonucleotides were used to target stem-cell-like cancer cells [77]. The miR-21 and miR-221 antisense oligonucleotides were given to sorted side population cells from gemcitabine-resistant cells and inhibited the growth and metastasis when both miRNAs were given. Schnittert et al. demonstrated a novel peptide-based nanocomplex for miR-199a antisense oligonucleotide delivery into human-derived PSCs [76]. The miR-199a antisense oligonucleotide inhibited PSC differentiation into cancer-associated fibroblasts and reduced the size of 3D heterospheroids made of PSCs and cancer cells. Taken together, these studies have shown that the combination of siRNA and chemotherapy not only has excellent anticancer effects, but also reduces toxicity and inhibits resistance.

## 4. Challenges and Future Prospects

Even with numerous efforts and ongoing investigations of RNAi-based therapeutics, pancreatic cancer remains as one of the most challenging with devastating malignancies with an extremely poor prognosis [165]. The successful delivery of degradation-prone nucleotide-based drugs is still the challenging goal in the field of RNAi therapeutics, requiring alternative strategies and reagents. One of the most promising mechanisms to consider in addressing the hurdles of intracellular RNAi delivery to gain cellular entry is macropinocytosis. The significance of macropinocytosis as a delivery pathway is that it is broadly applicable across multiple cell types, cargos, and delivery systems. Exploitation of macropinocytosis for therapeutic delivery to cancer cells is uniquely advantageous because macropinocytosis plays a key role in sustaining cancer cell growth within the nutrient-poor by functioning as a nutrient supply route [166,167,168,169,170]. Frequently observed oncogenic mutations, such as *KRAS, PI3K*, and *PTEN*, have been demonstrated to greatly enhance macropinocytosis in cancer cells [171,172,173,174]. Thus, it is not surprising to find that numerous delivery systems, such as lipids, polymers, and peptides, use macropinocytosis to enter a broad range of cancer cells [72,175,176,177,178,179,180]. Another important aspect of employing macropinocytosis in RNAi delivery is that macropinocytic entry delivers cargo to late endosomes and multivesicular bodies, which are particularly favorable destinations for nucleic acids [181,182,183]. Whether the siRNAs are delivered by lipids or nanoparticles, the intracellular trafficking of siRNAs begins in early endosomal vesicles [184]. The early endosomes subsequently fuse with sorting endosomes, which in turn transfer the content to the late endosomes. The acidic late endosomes later then relocated to the lysosomes where abundant nucleases exist. Thus, late endosomes and multivesicular bodies are where most endosomal escape is reported to occur before lysosomal degradation, a major barrier to the intracellular delivery of RNAi therapeutics [185]. These reports are especially interesting considering that cancer cells use macropinocytosis for nutrient scavenging, which requires lysosomal degradation of proteins into amino acids. The studies to elucidate the exact mechanism of macropinocytic delivery have encountered numerous difficulties due to the lack of specific inhibitors, varying rates among different cell types, and high sensitivity to external stimuli, and others [186,187]. However, our rapidly growing understanding of macropinocytosis presents unique opportunities to design better strategies to target macropinocytosis for intracellular delivery of RNAi.

One major hurdle specific to pancreatic cancer is the stromal barrier that hinders the therapeutic action of current and RNAi-based drugs within tumor cells or TME. Repeated reports and studies demonstrate that the tumor’s genetic aberrations cause the development of dense stromal environment and suppressive immune response, emphasizing the need for RNAi for TME reprogramming in addition to direct attack on tumor cells [188]. Targeting the infiltrated immune cells present in stromal structure is an alternative approach to cancer therapy using immunotherapeutic options. The use of nanoparticles has led to successful delivery of RNAi drugs into tumor cells; however, the uptake rate remains low for clinical significance. Similar to the immunotherapeutic drug agents, RNAi has the potential to take action on immune cells to re-sensitize the suppressive immune response as an alternative cancer therapeutic strategy [189]. Genetic mutations of signaling proteins and transcription factors triggering the immunosuppression are potential RNAi drug targets with benefits over chemical inhibitors and conventional therapies. Furthermore, the application of RNAi drugs with current checkpoint inhibitors has great potential in clinical translation to patients who developed resistance to immunotherapy.

Although milestones of RNAi-based drugs have proved clinical translation of oligo therapeutics and has opened numerous application possibilities, the systemic delivery challenge remains for specific disease targets. When designing a delivery strategy for oligonucleotide, the cargo and moiety of drug often jeopardizes the safety profile and cause adverse toxicities upon accumulation. Though RNAi-based drugs are naturally occurring biological materials, the chemical modifications and fabrication is inevitable and accompanies safety concerns. For this reason, strategies to use siRNA conjugation with more transparent and simpler structures and designs is under investigation across academia and industry for its advantages in clinical translation.

## 5. Conclusions

The potential to regulate selective gene expression using RNAi-based moieties holds promise for pancreatic cancer treatment. Numerous results demonstrating antitumor effects, chemosensitization, anti-metastatic activity and activation of immunogenicity against cancer cells have been shown in preclinical and even in early clinical stages. Pancreatic cancer, one of the cancer types with the lowest survival rate, is uniquely identified with dense tumor microenvironment serving as a barrier to conventional drugs. Dysregulated expression of cancer-associated genes and key signaling pathways are often the driver for acquired resistance and the discouraging outcomes of current therapies. For this reason, the RNAi-based approaches have gained attention to elicit antitumor effect or altering the nature of TME to remove barriers for chemotherapeutic action. From the studies mentioned above, it appears that RNAi-based drugs hold great hope in pancreatic cancer therapy [190]. In addition to the RNAi-based drug discovery and development, it is crucial to develop a model or system that accurately reflects the biology of human pancreatic cancer with clinically similar features. Orthotopic pancreatic cancer mouse models with an intact and functional fibrotic stroma, and genetically engineered mouse models with functional immune system and 3D tissue models have been developed and are continually being improved to advance pancreatic cancer therapy [191]. In particular, the use of 3D cultures is an innovative approach that narrows the gap between traditional 2D cell culture and animal models allowing the manipulation of individual cell population involved in TME specific to the disease type [192]. Several groups have demonstrated spheroids, hydrogel structures, and microfluidic designs to mimic the TME nature of pancreatic cancer [193,194,195,196,197]. These models, used in parallel with clinically relevant in vivo models, can inform the design of novel RNAi-based drugs. Since personalized medicine and targeted therapies are the next generation of cancer treatments, the unique pathophysiology of pancreatic cancer needs to be addressed. In particular, the dense fibrotic stroma presenting a large barrier is a strong consideration in introducing a novel RNAi-based approach for pancreatic cancer therapy. Despite the heterogeneity and complexity of pancreatic cancer, we believe that versatility of RNAi-based drugs with numerous targets hold promising potential for pancreatic cancer therapy in combined with chemotherapy or immunotherapy.

## Figures and Tables

**Table 1:** Various models of pancreatic cancer for preclinical studies.

	In Vitro		In Vivo		
Models	Cell Line	Patient-Derived Organoid	Cell Line Xenograft	Patient-Derived Xenograft	Genetically Modified Mouse Model (GEMM)
TME	-	+++	+	++	++
Immune system	-	++	+	+	+++
Pros	Fast and easy growth Rapid drug screening	Mimic complex TME and stroma Orthotopically transplantable	Useful for testing drug efficacy and safety	Mirror patient response to drug Personalized drug regimen	Useful tool for oncogenic mutation investigation and biomarker discovery
Cons	Genetically uniform	New model and needs further analysis	Limited TME and immune system	Engraftment difficulties Long duration of growth Selection for aggressive tumors	Species discrepancy High cost and complexity of GEMM generation

Parameters are appreciated as abundant (+++), moderate (++), minimal (+) and none (-).

**Table 3:** miRNAs used for preclinical pancreatic cancer therapy.

miRNA	Target	PC Cell Line/Model	Combination Therapy	Reference
miR-150	MUC4 and HER2	Colo-357 and HPAF cells		[74]
miR-155	SOD2, CAT, and DCK	MiaPaCa and Colo-357 cells	Gemcitabine	[75]
miR-199a ASO	RPS18, Acta-2, Collagen1α1, PDGFR-β, and mTOR	hPSCs		[76]
miR-21 and miR-221 ASO	CDK6, IRAK3, NRP1, SMAD7, SOCS6, C5ORF41, KLF12, MAPK10, EFNA1	L3.6plGres-SP orthotopic		[77]
miR-21 ASO	PDCD4 and PTEN	MIA PaCa-2 s.c.	Gemcitabine	[78]
miR-212	USP9X	PDX	Doxorubicin	[79]
miR-345	SHH, Gli-1, MUC4, and Ki67	Capan-1 and CD18/HDAF s.c.	Gemcitabine	[80]
miR-34a	E2F3, Bcl-2, c-myc, and cyclin D1	PANC-1 s.c.		[81]
miR-34a and miR-143/145	SIRT1, CD44, aldehyde dehydrogenase, KRAS2, and RREB1	MiaPaCa-2 s.c. and orthotopic		[82]

Abbreviations: miR, microRNA; ASO, antisense oligonucleotide; MUC4, mucin 4; HER2, human epidermal growth factor receptor 2; SOD2, superoxide dismutase 2; CAT, catalase; DCK, deoxycytidine kinase; RPS18, ribosomal protein S18; Acta-2, actin alpha 2; PDGFR, platelet-derived growth factor receptor; mTOR, mechanistic target of rapamycin; CDK6, cell division protein kinase 6; IRAK3, interleukin 1 receptor associated kinase 3; NRP1, neuropilin 1; SMAD7, SMAD family member 7; SOCS6, suppressor of cytokine signaling 6; C5ORF41, chromosome 5 open reading frame 41; KLF12, Krueppel-like factor 12; MAPK10, mitogen-activated protein kinase 10; EFNA1, Ephrin-A1 precursor; PDCD4, programed cell death protein 4; PTEN, phosphatase, and tensin homolog; USP9X, ubiquitin specific peptidase 9 x-linked; SHH, sonic hedgehog; Bcl-2, B-cell lymphoma 2; SIRT1, sirtuin 1; RREB1, ras responsive element binding protein 1; s.c., subcutaneous; PDX, patient-derived xenograft.
